# Evaluating the biomedical and behavioral drivers of HIV incidence decline in adolescent girls and young women in Uganda: A mathematical modeling study

**DOI:** 10.1371/journal.pmed.1004993

**Published:** 2026-03-25

**Authors:** Adam Akullian, Victor Ssempijja, Daniel Bridenbecker, Fred Nalugoda, Gertrude Nakigozi, John Santelli, Philip Kreniske, Larry W. Chang, Steven J. Reynolds, Robert Ssekubugu, Ronald H. Gray, Maria J. Wawer, Thomas C. Quinn, Ronald M. Galiwango, William J. M. Probert, Jeffrey W. Imai-Eaton, Oliver Ratmann, Christophe Fraser, Joseph Kagaayi, Godfrey Kigozi, Mary Kate Grabowski, David Serwadda

**Affiliations:** 1 Institute for Disease Modeling, Gates Foundation, Seattle, Washington, United States of America; 2 Clinical Monitoring Research Program Directorate, Frederick National Laboratory for Cancer Research, Frederick, Maryland, United States of America; 3 Rakai Health Sciences Program, Kalisizo, Uganda; 4 Population and Family Health and Pediatrics, Columbia University, New York, New York, United States of America; 5 Graduate School of Public Health and Health Policy, City University of New York, New York, New York, United States of America; 6 Johns Hopkins School of Medicine and Bloomberg School of Public Health, Baltimore, Maryland, United States of America; 7 Division of Intramural Research, National Institute of Allergy and Infectious Diseases, National Institutes of Health, Bethesda, Maryland, United States of America; 8 Department of Medicine, Johns Hopkins University School of Medicine, Baltimore, Maryland, United States of America; 9 University of Oxford, Oxford, United Kingdom; 10 Center for Communicable Disease Dynamics, Department of Epidemiology, Harvard T.H. Chan School of Public Health, Boston, Massachusetts, United States of America; 11 MRC Centre for Global Infectious Disease Infectious Disease Analysis, School of Public Health, Imperial College London, London, United Kingdom; 12 Department of Mathematics, Imperial College London, London, United Kingdom; 13 Li Ka Shing Centre for Health Information and Discovery, University of Oxford, Oxford, United Kingdom; 14 Departments of Pathology and Epidemiology, Johns Hopkins University, Baltimore, Maryland, United States of America; 15 Makerere University School of Public Health, Rakai Health Sciences Program, Kalisizo, Uganda; University of Ottawa, CANADA

## Abstract

**Background:**

HIV incidence among adolescent girls and young women (AGYW) in eastern and southern Africa has declined substantially over the past two decades. These declines are often attributed to biomedical HIV prevention strategies, though concurrent changes in sexual behavior may also contribute. We evaluated the contributions of biomedical and behavioral drivers to historical incidence decline in AGYW and projected their impact on incidence trajectories over the next 30 years.

**Methods and findings:**

We conducted a mathematical modeling study using data from the Rakai Community Cohort Study (RCCS), an open, population-based cohort of adults aged 15–49 years in 30 communities in Rakai, Uganda. We used an agent-based HIV-1 transmission model calibrated to cohort data to estimate HIV incidence trends among AGYW, aged 15–24, and to quantify the independent and combined effects of antiretroviral therapy (ART), voluntary medical male circumcision (VMMC), and changes in age at first sex (AFS).

HIV incidence among women aged 15–24 declined by 71% between 2000 and 2019, from 1.57 to 0.45 per 100 person-years, representing the largest decline across female age groups in the cohort. Increasing AFS over the study period (by approximately 3 years in women and 2 years in men) was the largest contributor to incidence declines among adolescent women aged 15–19, averting 17% of cumulative infections between 2000 and 2020 and 37% between 2000 and 2050. Among women aged 20–24, ART scale-up had the greatest impact, averting 13% of infections by 2020 and 43% by 2050. VMMC contributed modestly to historical declines but had larger projected effects over longer time horizons. ART, VMMC, and delays in AFS acted additively to reduce HIV incidence among AGYW. Study limitations include reliance on self-reported sexual behavior and the use of a mathematical model that cannot capture all real-world sexual network dynamics.

**Conclusions:**

Both biomedical HIV interventions and broader behavioral changes contributed to declines in HIV incidence among AGYW. Sustaining continued incidence declines in young women will require maintaining both the protective changes in sexual behaviors and effective biomedical interventions.

## Author summary

**Why was this study done?:**

- Adolescent girls and young women in eastern and southern Africa have historically faced the highest risk of acquiring HIV.
- Over the past two decades, HIV infections among young women in Uganda have declined sharply, but the reasons for this decline are not fully understood.
- Both biomedical interventions (such as HIV treatment and male circumcision) and changes in sexual behavior (such as later age at first sex) occurred during this period, making it important to understand their individual and combined effects.

**What did the researchers do and find?:**

- We used a mathematical model calibrated to long-term population data from southern Uganda to examine changes in HIV incidence between 2000 and 2019.
- HIV incidence among young women aged 15–24 declined by more than 70%, with the largest reductions occurring among adolescents aged 15–19.
- Delays in the age at which young people first had sex, along with expanded access to HIV treatment and male circumcision, each contributed to these declines, and their effects were largely additive.

**What do these findings mean?:**

- Our results show that both biomedical HIV interventions and broader social and behavioral changes have played important roles in reducing HIV infections among young women.
- Sustaining progress toward HIV epidemic control will likely require continued investment in HIV treatment and prevention alongside efforts that support education and social conditions associated with delayed sexual debut.
- This study relied on self-reported sexual behavior and mathematical modeling, which may not capture all real-world complexities, and future changes in HIV programs or social conditions could alter long-term projections.

## Abstract 3

Using mathematical modeling, Adam Akullian and colleagues estimate that increasing age at first sex is a key driver of the decline in HIV incidence among young women in the Rakai Community Cohort Study in Uganda.

## Introduction

Adolescent girls and young women (AGYW) in east and southern Africa have experienced among the highest rates of HIV acquisition in the world [1–3]. Several behavioral, biological, and socio-economic factors put young women at disproportionately high risk of HIV acquisition [4,5]. These factors include higher per-sex-act risk of acquisition from increased biological susceptibility to HIV infection [6,7], limited ability to negotiate safer sex and condom use [8], poverty and school dropout [3–5], gender-based violence [9], and higher prevalence of unsuppressed viral load in older male partners [10–12].

Uganda has experienced substantial declines in HIV incidence and prevalence since the late 1990s, driven by early national responses [13] and later expansion of biomedical interventions such as voluntary medical male circumcision (VMMC) and widespread antiretroviral therapy (ART) coverage [14,15]. The HIV epidemic remains generalized, with marked geographic and age/sex heterogeneity; incidence tends to be highest among young women and in high-burden districts along major transport corridors and fishing communities [16].

Over the last decade, HIV incidence has declined substantially in AGYW across communities in southern and eastern Africa [17,18], especially compared to that of older women, whose incidence has remained relatively stable in certain settings [18–21]. Multiple factors, including the scale-up of biomedical interventions [18,22–25] and reductions in sexual behaviors that increase HIV risk [19], are likely contributors to incidence decline among AGYW [26], but no study has disentangled the independent effects of these hypothesized drivers.

Age at first sex (AFS) marks the initiation of exposure to sexually transmitted infections (STIs), including HIV, and is associated with alarmingly high HIV risk in young African women [27]. Earlier initiation of sex is associated with higher risk of HIV acquisition [9,28–32] via several socio-behavioral [9] and biological [33] pathways. Consequently, delaying sex protects against HIV infection by reducing the time spent sexually active, lowering the overall number of sexual partners, and reducing biological and behavioral susceptibility to infection. Rising AFS in young adults has been observed across sub-Saharan Africa [34], driven by several social and structural factors, including rising socio-economic status (SES) and expanded educational opportunities for adolescents [35,36]. It is unclear, however, to what extent recently observed delays in AFS in young adults contribute to population-level declines in incidence among AGYW in the context of large-scale expansion of biomedical interventions like ART and VMMC.

Because biomedical and behavioral drivers act on HIV risk via conceptually distinct pathways, understanding their relative contributions has programmatic importance for funding and intervention prioritization. Biomedical interventions directly reduce biological susceptibility and infectiousness, while behavioral shifts modify the probability of exposure. These components can act synergistically, where, for example, reductions in partner numbers amplify the population-level impact of ART-driven decreases in community viral load [37], but may also operate redundantly when they target similar segments of the population.

Here, we use a mathematical model calibrated to data from the Rakai Community Cohort Study (RCCS), a population-based cohort in southern Uganda, to quantify the independent and combined contributions of ART, VMMC, and secular changes in AFS to historical and projected declines in HIV incidence among AGYW.

## Methods

### Study data

The RCCS is one of the longest-standing population-based HIV surveillance programs, following an open cohort of adolescents and adults (15–49 years of age) from 30 continuously surveyed communities over a 20-year period between 1999 and 2019 (with continued follow-up to date). Approximately 40,000 adult residents enrolled in the RCCS, who were present for at least one survey round, provided data over the length of follow-up. While the focus of our study is on AGYW, we include all adults 15–49 in our analysis to provide epidemiological context for our results.

Details of the RCCS and survey methods are found elsewhere [19,38]. Briefly, participants 15–49 years of age from a complete household census of 30 semi-urban and agrarian communities in four districts of southern Uganda were invited to participate in the longitudinal study. Participants were recruited into the open cohort throughout the study period and those who provided written informed consent were interviewed every 1–2 years between 1999 and 2019, constituting 14 survey rounds. Adolescents 15–17 years of age required assent with parental consent. Self-reported data on demographics, SES, sexual risk behaviors, partnership attributes, ever ART use, and circumcision status (for men), were collected at each survey round. Sexual behavior was captured through interviewer-administered surveys in private settings. Core questions (e.g., AFS, partnership status) remained consistent over time. HIV testing was offered to all adult participants (ages 15–49), and venous blood was obtained from those consenting to HIV testing (including 15–17-year-olds who assented with parental consent). Laboratory methodology for HIV testing is described elsewhere [19]. The analysis was restricted to residents at the time of survey. The mean participation rate among adults eligible for the survey ranges from 59% to 67% across survey rounds, with most non-participation due to an individual not being in the community at time of survey. Uptake of HIV testing among those reached is high across survey rounds [21]. Participants in the study completed, on average, 3.5 survey rounds. Self-reported ART coverage (percent of respondents who report ever having been on ART) was found to be similar in models adjusted for attributes associated with survey response [21], indicating low potential for selection bias.

### Estimation of key outputs

HIV incidence was estimated from directly observed seroconversion among ~20,000 participants with an HIV–negative test followed by at least one HIV test during a subsequent survey round. For those testing positive at a subsequent survey round, a seroconversion round was imputed randomly between the last negative and the first positive testing round, a method that reduces precision but introduces minimal bias into estimates [39]. Follow-up was right censored at either the seroconversion round (for those testing positive) or last negative test round (for those who remained serially negative). Individuals with missing HIV status between two non-missing survey rounds with the same status (e.g., two negatives or two positives) had their missing HIV status filled in using the two non-missing rounds. For example, an individual with positive status at rounds 6 and 10 and missing status in between had positive status filled in for rounds 7, 8, and 9, with a similar method applied to those with missing test data between two negative tests. We excluded records where the first observed positive was on/before the last negative (i.e., no valid seroconversion window), which occurred rarely in the dataset.

Person-years of follow-up and crude numbers of incident infections were calculated for each survey round for individuals included in the incidence cohort. Smoothed Poisson generalized additive models (GAMs) [40] with an offset for person-years at risk [41] were used to estimate sex-specific smoothed incidence rates and 95% confidence intervals. Age and survey round were specified as a smooth function with a main effect and interaction. GAMs were fit with penalized, thin plate regression splines [40], (isotropic, locally weighted regression smoothers). The expected number of seroconversions ${E(y}_{i})\;$ was estimated as the product of the Poisson-distributed seroconversion rate $\lambda_{i}$ and person-time at risk $T_{i\;}$. Linear combinations of 1- and 2-dimensional smooth terms, $f_{\text{1},\ldots ,}f_{p}$ were included as main effects in the model:


$$y_{i}\;\sim \text{ Poisson}\left(\lambda_{i}T_{i}\right)=$$

$$log\;[\beta_{\text{0}}+f_{\text{1}}\left({\text{age}}_{i\;}\right)+f_{\text{2}}\left({\text{round}}_{i\;}\right)+f_{\text{3}}\left({\text{age}}_{i\;},{\text{round}}_{i\;}\right)+T_{i}+\;\varepsilon_{i}],$$

where$\;\beta_{\text{0}}\;$ is an intercept, $\varepsilon_{i\;}$ are identically distributed and independent (i.i.d.) errors, and $f_{\text{1},\ldots ,}f_{p}$ are smooth regression splines, defined as the sum of piece-wise polynomial basis functions $b_{j}$ and regression coefficients $Z_{j}$ with basis dimension J:


$$\begin{array}{c} f(x)=\sum_{j\;\;=\;\;\text{1}}^{J}b_{j}(x)Z_{j} \end{array}$$

Generalized linear models (GLMs) were also fit with categorical terms for survey round and age group to estimate sex, age group, and round-specific incidence rates and 95% confidence bounds to compare with smoothed GAMs.


$$y_{i}\;\sim \text{ Poisson}\left(\lambda_{i}T_{i}\right)=log\left[\beta_{\text{0}}+\beta_{\text{1}}\left({\text{age group}}_{i\;}\right)+\beta_{\text{2}}\left({\text{round}}_{i\;}\right)+\beta_{\text{3}}\left({\text{age group}}_{i\;}\times {\text{round}}_{i\;}\right)+T_{i}+\;\varepsilon_{i}\right].$$

HIV prevalence was estimated cross-sectionally at each survey round using binomial GAMs with a logit link function:


$$y_{i}\;\sim \text{ Binomial}\left(p_{i}\right)=\text{logit}\left[\beta_{\text{0}}+f_{\text{1}}\left({\text{age group}}_{i}\right)+f_{\text{2}}\left({\text{round}}_{i\;}\right)+f_{\text{3}}\left({\text{age group}}_{i\;}\times \;{r\text{ound}}_{i\;}\right)+T_{i}+\;\varepsilon_{i}\right].$$

ART coverage (% on ART), male circumcision coverage (% circumcised), and prevalence of having initiated sex (% reporting ever having sex), were similarly estimated cross-sectionally at each survey round for all individuals with non-missing data on each indicator using binomial GAMs with the sames specification as the HIV prevalence models.

### Mathematical modeling

Micro-simulation modeling was implemented using the epidemiological modeling software EMOD-HIV, an agent-based, stochastic, network transmission model described in detail at https://docs.idmod.org/projects/emod-hiv/en/latest and elsewhere [42]. EMOD simulates the natural history of HIV progression, considering the variable health states of individuals, such as CD4 cell count and WHO disease stage. A diagram of model structure can be found at https://docs.idmod.org/projects/emodpy-hiv/en/latest/emod/hiv-model-overview.html.

EMOD uses data on age-specific fertility, mortality, and sexual relationship formation [43], and models the cyclical flow of people living with HIV through the HIV care cascade. ART and VMMC were allocated based on age, sex, and year-specific coverage estimates from cohort data. We estimated a per-contact ART efficacy of 92% [44], to reflect “real-world” barriers to suppression like adherence and undetected drug resistance. Other factors in our model that modify the transmission probability include condom usage, circumcision status of the male HIV–negative partner, age of the female HIV–negative partner (to capture increased biological susceptibility of younger women), and presence of an STI.

EMOD simulates the age, gender, and risk-group-specific sexual network using a sexual partner pair formation algorithm (PFA) described previously [43]. The PFA provides a detailed representation of the age-specific structuring of heterosexual networks—a phenomenon that contributes to the age- and gender-specific patterns of HIV transmission [27,43]. We capture the effects of delayed age at sexual debut on relationship formation by matching to survey data the percentage of individuals by age and sex available to form relationships on the network. In this way, changes in the proportion of individuals reaching sexual debut over time affect both the person-time exposed to HIV and the biological risk of acquisition among those delaying sex to older ages. The PFA allows redistribution of partnerships within the network and therefore partnership replacement can occur in response to changes in sexual debut. In this way, older male partners who would otherwise form relationships with younger women instead form partnerships with similarly aged women.

Models were calibrated to age-, gender-, and year-specific HIV prevalence and age-, gender-, and year-specific population numbers from the RCCS between 1999 and 2019. Cohort-based estimates of ART coverage, VMMC coverage, and proportion having initiated sex by age, sex, and round were specified as modeled inputs. Pre-exposure prophylaxis (PrEP) was not included in the model because of its limited availability in the population and the uncertainty around its future scale-up in the population. A total of 100 best-fitting parameter sets were sampled from 6,600 simulations using roulette resampling in proportion to the likelihood. Uncertainty was incorporated into modeled estimates using a range of model parameters calibrated to observed epidemiologic trends. A summary of calibrated and static model parameter values and descriptions can be found in (Tables B and C in S1 File).

### Model scenarios and outcomes

For historical (2000–2020) and future projections (2000–2050), we modeled HIV incidence per 100 person-years, cumulative infections averted, and cumulative % of infections averted. We compare several counter-factual scenarios to a baseline scenario, which captures the historical scale-up of ART/VMMC and the observed increases in AFS between 2000 and 2019. The four counterfactual scenarios are as follows: (1) a scenario with no ART scale-up (ART coverage set at zero), (2) a scenario with no VMMC scale-up (VMMC coverage set at zero), (3) a scenario with no changes in AFS (set at baseline levels estimated at the start of the cohort study), and (4) a scenario with no interventions (including no changes in AFS). For each scenario, we held levels of each intervention and AFS constant from 2020 to 2050 for projected impacts.

### Ethics

Ethical approval for the RCCS was obtained from the Uganda Virus Research Institute’s Research Ethics Committee (No: GC/127/19/11/137) and the Uganda National Council for Science and Technology (HS 540). Approval was also obtained from IRBs of collaborating institutions, including the Committee for Human Research at the Johns Hopkins University School of Public Health and School of Medicine (JHU NA_00069085) and the Western University Institutional Review Board. All participants provided written informed consent for the broader protocol under the RCCS.

## Results

### Study population

The population of adult residents ages 15–49 enrolled in the RCCS who participated in at least one of the 14 survey rounds between 1999 and 2019 included 40,800 participants (17,800 men and 23,000 women). Mean age at first survey visit was 23.7 years in women and 24.1 years in men. Among women 15–24 included in the analysis of AGYW, mean age at first survey visit was 18.8 years, 48.3% had ever been married, mean AFS was 15.7 years, and mean number of sex partners among those sexually active in the last year was 1.2.

### HIV incidence

The cohort providing data to estimate HIV incidence (those with a negative test followed by a subsequent test) included 22,017 participants aged 15–49, among whom 1,317 incident infections were observed (795 in women and 522 in men) in 141,537 person-years of follow-up, (76,056 person-years of follow-up in women and 65,481 person-years of follow-up in men), resulting in a crude incidence rate of 0.93/100 py (0.88–0.98) overall, and 1.05/100 py (0.97–1.12) in women and 0.80/100 py (0.73–0.87) in men over the 20-year study period. A full enumeration of incident infections, person-years, incidence/100py, and 95% confidence intervals (CIs) by age group and year for both men and women can be found in Table A in S1 File.

**Fig 1:**
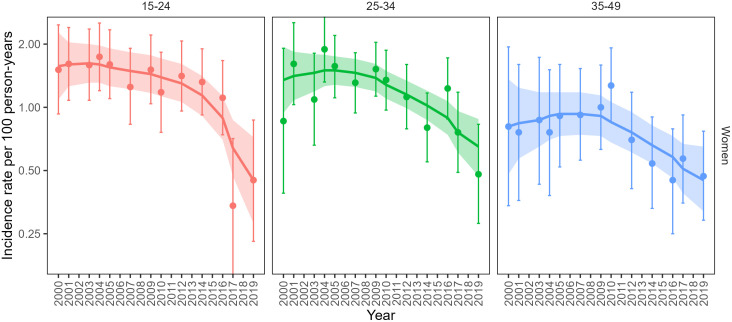
Age-specific HIV incidence in the Rakai Community Cohort Study over time (2000-2019) among women 15–49, estimated from cohort data. Point estimates and 95% confidence intervals (error bars) estimated from categorical generalized linear models (GLMs); smoothed incidence curves and 95% confidence intervals (ribbons) estimated from smoothed generalized additive models (GAMs). Note incidence is on a log2 scale.

**Fig 2:**
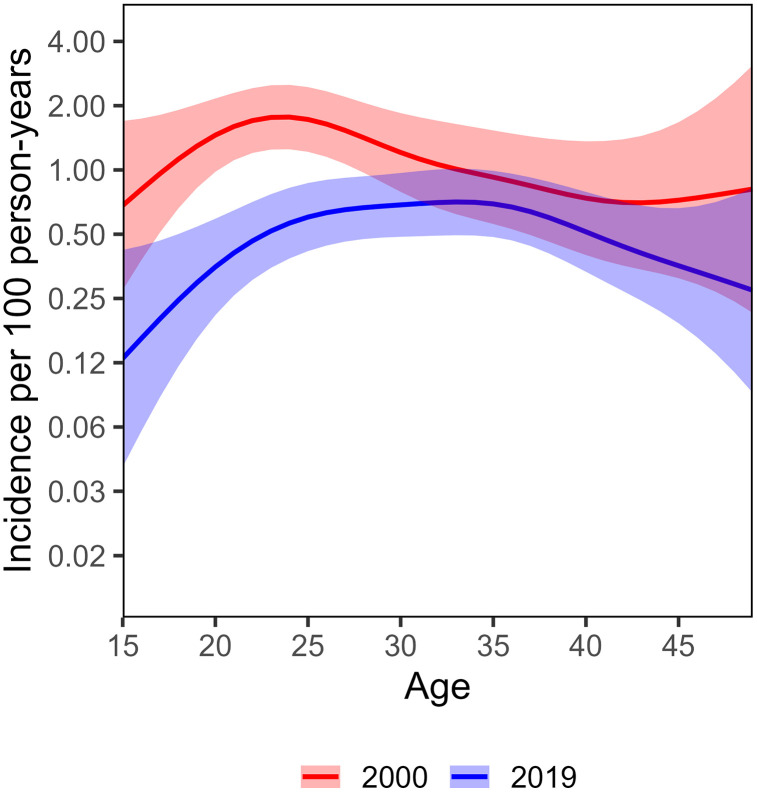
Smoothed continuous age distributions of HIV incidence among women 15–49 comparing the first (year 2000) and last (year 2019) survey rounds. Incidence estimated from cohort data using smoothed generalized additive models (GAMs). Note incidence is on a log2 scale.

Among women 15–24 years of age, 283 incident infections were observed in 22,827 person-years, a crude incidence rate of 1.24 per 100 py (1.10–1.39) (Fig 1). Based on smoothed GAM models, HIV incidence in women 15–24 declined by 71% (from 1.57 per 100 py in 2000 to 0.45 per 100 py in 2019), with most of the decline occurring after 2014 and a marked drop between 2016 and 2017. Older age groups of women also experienced declines in incidence, though less than that of the youngest women: incidence declined by 52% among women 25–34 (from 1.35 per 100 py in 2000 to 0.65 per 100 py in 2019) and by 44% among women 35–49 (from 0.81 per 100 py in 2000 to 0.45 per 100 py in 2019). The larger relative decline among younger versus older women resulted in a shift toward an older incidence age distribution in 2019 compared with 2000, with the highest incidence shifting from the 15–24 group in 2000 to the 25–34 group in 2019 (Fig 2). The age group with the highest incidence increased by about 10 years over the 20-year study period, peaking among women 20–24 years in 2000 and among women 30–34 years of age in 2019.

### ART coverage, VMMC coverage, and percent ever initiating sex

**Fig 3:**
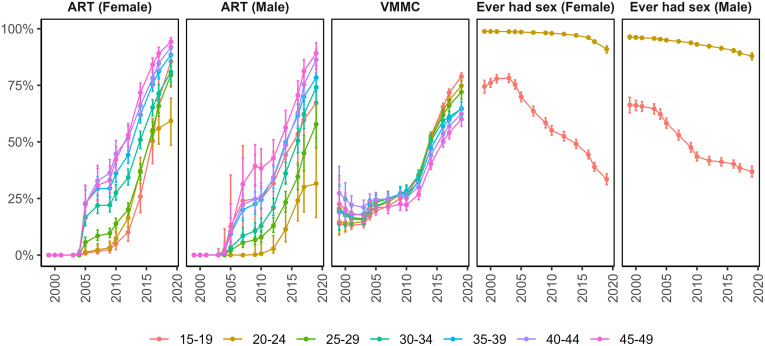
Antiretroviral (ART) coverage (by age-group and sex), voluntary medical male circumcision (VMMC) (by age-group), and prevalence of having initiated sex among 15–19 and 20–24 (by sex). Point estimates and 95% confidence bounds estimated from smoothed generalized additive models (GAMs) with an interaction between year and age-group.

Fig 3 shows temporal trends in ART coverage (% on ART), VMMC coverage (% circumcised), and the % of young men and women who reported ever initiating sex. ART began to scale-up after 2004 and steadily increased to 2019 and increased earlier and to higher coverage in older men and women. By 2019, the highest coverage was in women 45–49 years of age, 94% (92%–96%) and the lowest ART coverage in men 20–24 years of age, 32% (17%–52%).

The percentage of circumcised men increased steadily over the study period. VMMC scaled up slowly from 1999 to 2009, after which it climbed more steeply to 2019. VMMC coverage was similar across all ages prior to 2010 and increased more rapidly in younger men thereafter. By 2019, 80% (79%–82%) of men 15–19 were circumcised compared to 60% (56%–63%) of men 45–49 years of age (Fig 3).

The proportion of men and women 15–24 years of age who reported ever having initiated sex declined over the course of the study. Among women 15–19 years of age, half as many reported having initiated sex in 2019 compared to 1999 (from 80% to <40%). Similar declines were reported by young men. By 2019, similar proportions of young men and women reported having initiated sex (Fig 3).

### Mathematical modeling

**Fig 4:**
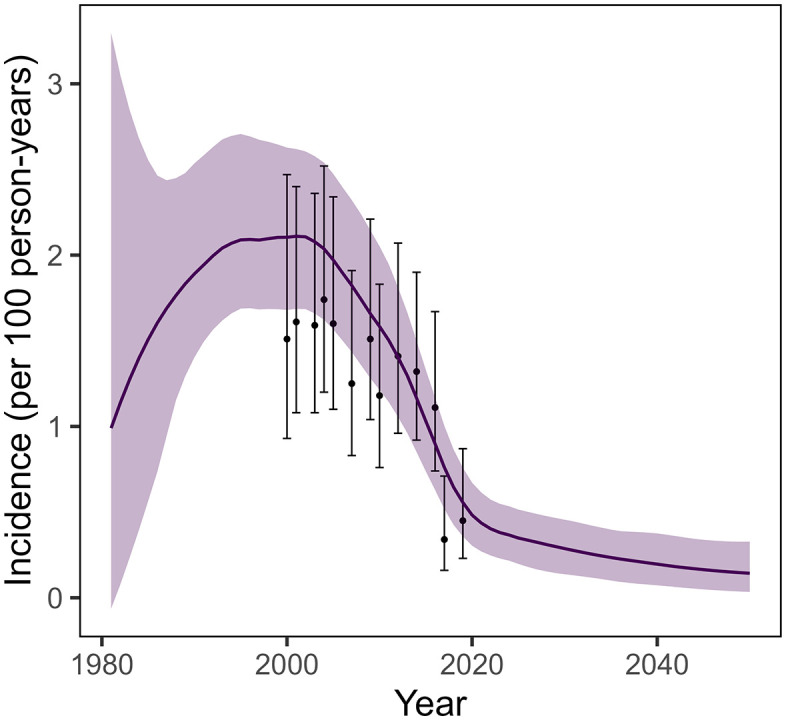
Comparison of modeled HIV incidence and estimated yearly incidence observed in the Rakai Community Cohort Study (RCCS) among women 15–24 years of age. Purple curve with ribbon shows range of 100 simulations from modeled incidence.

Calibrated model fits to observed HIV prevalence by age, sex, and year are shown in (Figs A and B in S1 File). Modeled HIV incidence among women 15–24 years of age had a similar trend between 2000 and 2020 as that of directly observed incidence estimated from the RCCS, though modeled estimates tended to be slightly higher than observed incidence (Fig 4). Modeled incidence declined from 2.1/100 py (1.6–2.6/100 py) in year 2000 to 0.47/100 py (0.29–0.64/100 py) in year 2020 and is projected to continue to decline to 0.2/100 py (0.07–0.39/100 py) by 2040 assuming stable rates of ART, VMMC, and AFS over that time horizon.

**Fig 5:**
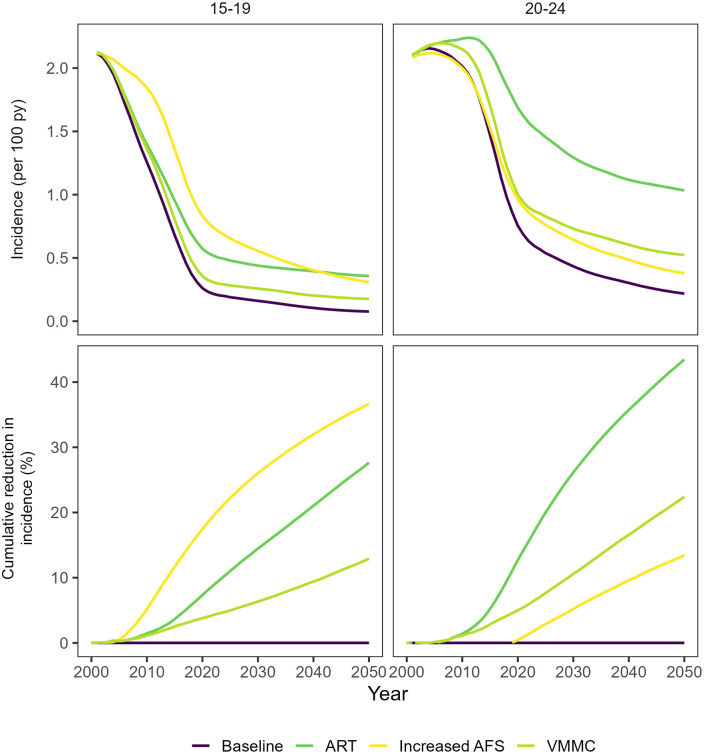
Modeled counterfactual scenarios compared to baseline among women 15-24. (Top panel) mean incidence rates and (bottom panel) mean cumulative infections averted among women 15–19 and 20–24 by scenario. The percentage of cumulative infections averted is the percent reduction in cumulative infections between the counterfactual scenario and the baseline scenario.

**Fig 6:**
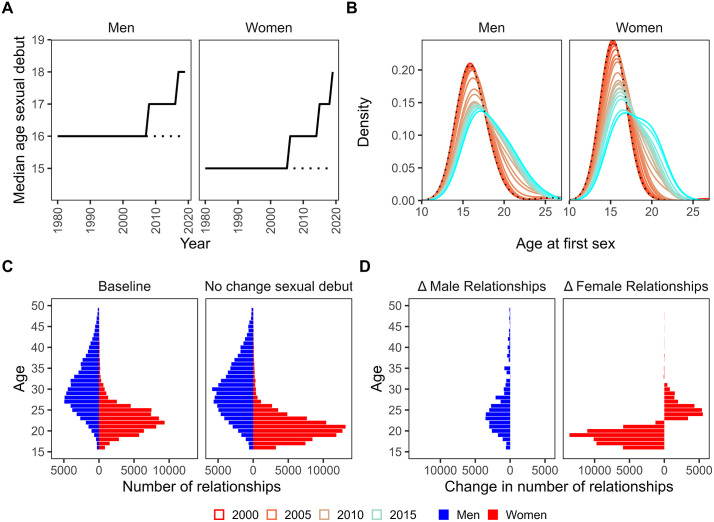
Modeled age at first sex and changes in numbers of partners from baseline. **(a)** Median age at first sex (AFS) by year in the baseline scenario (solid black lines) compared to no change in AFS scenario (dotted lines). **(b)** Distribution of AFS by year. **(c)** Crude number of sexual relationships formed by age, sex, and scenario (baseline vs. no change in AFS). **(d)** Difference in crude number of sexual relationships formed between baseline scenario and no change in AFS scenario. Numbers to the left of zero indicate fewer relationships formed in the baseline scenario and numbers to the right of zero indicate more relationships in the baseline scenario relative to the no change in AFS scenario.

HIV incidence and cumulative infections averted are shown over time for women aged 15–19 and 20–24 for each counterfactual scenario relative to the baseline scenario (Fig 5). Modeled increases in AFS (by 2 years in men and 3 years in women) (Fig 6a) had the largest effect on incidence in women 15–19 years of age, averting 17% of cumulative infections between 2000 and 2020 and 37% of cumulative infections between 2000 and 2050 (Fig 5). The incidence rate among 15–19 year olds in 2020 was 69% lower in the baseline, 0.26/100 py (0.15–0.40/100 py) versus the counterfactual scenario with no change in AFS, 0.83/100 py (0.51–1.27/100 py), and in 2050 the incidence rate is projected to be 75% lower in the baseline, 0.08/100 py (0.01–0.17/100 py) versus the counterfactual scenario with no change in AFS, 0.31/100 py (0.08–0.77py) (Table 1). Among women 20–24 years of age, increases in AFS had no effect on cumulative incidence between 2000 and 2020, and averted 13% of cumulative infections by 2050. In women 20–24 years of age, incidence in 2050 is projected to be 45% lower in the baseline scenario, 0.21/100 py (0.05–0.53/100 py) compared to the AFS counterfactual scenario, 0.38/100 py (0.11–0.73/100 py) among women 20–24 years of age.

**Table 1:** Modeled age and year-specific incidence rates by scenario among women 15-24.

Scenario	Age	Year	Incidence per 100 py		
			Mean	lb	ub
Baseline	15–19	2020	0.26	0.15	0.40
		2050	0.08	0.01	0.17
	20–24	2020	0.73	0.44	1.01
		2050	0.21	0.05	0.53
No ART	15–19	2020	0.57	0.38	0.79
		2050	0.36	0.19	0.55
	20–24	2020	1.66	1.13	2.29
		2050	1.03	0.54	1.54
No change in AFS	15–19	2020	0.83	0.51	1.27
		2050	0.31	0.08	0.77
	20–24	2020	0.95	0.55	1.33
		2050	0.38	0.11	0.73
No VMMC	15–19	2020	0.36	0.22	0.56
		2050	0.17	0.05	0.33
	20–24	2020	0.96	0.62	1.32
		2050	0.52	0.16	0.92
No Interventions	15–19	2020	2.31	1.60	3.27
		2050	2.70	1.65	3.98
	20–24	2020	2.61	1.65	3.71
		2050	3.09	1.63	4.63

Mean and 95% credible intervals. Antiretroviral therapy (ART), age at first sex (AFS), voluntary medical male circumcision (VMMC)

ART scale-up to date had a more moderate effect on incidence in women 15–19 years of age and the largest effect of any intervention on incidence in women 20–24 years of age. ART scale-up averted 7% of infections in women 15–19 years of age and 13% of infections in women 20–24 years of age between 2000 and 2020. By 2050, ART scale-up is projected to avert 28% of infections in women 15–19 years of age and 43% of infections in women 20–24 years of age. In 2020 incidence in women 15–19 years of age was 54% lower in the baseline scenario, 0.26/100 py (0.15–0.40/100 py) compared to the scenario without ART, 0.57/100 py (0.38–0.79/100py), and by 2050 incidence in women 15–19 years of age is projected to be 78% lower in the baseline, 0.08/100 py (0.01–0.17/100 py) compared to incidence in the scenario without ART, 0.36/100 py (0.19–0.55/100 py). VMMC scale-up to date (Fig 3) had a small impact on incidence reduction in young women to date, averting 4% of cumulative infections in women 15–19 years of age and 5% of cumulative infections in women 20–24 years of age between 2000–2020, and a larger impact over longer time horizons: 13% of cumulative infections averted in women 15–19 years of age and 22% of cumulative infections averted in women 20–24 years of age between 2000 and 2050.

Combined, all three scenarios (ART, VMMC, and increased AFS) reduced cumulative infections by 32% in women 15–19 years of age and by 19% in women 20–24 years of age between 2000 and 2020 and reduced cumulative infections by 74% in women 15–19 years of age and 65% in women 20–24 years of age between 2000 and 2050. The combined effect of the three scenarios when modeled together was similar to the sum of the individual effects of each scenario for women 15–19 years of age over both time horizons and for women 20–24 years of age between 2000 and 2020, indicating largely additive effects of both interventions and increases in AFS. For women 20–24 years of age between 2000 and 2050, the combined effect for all scenarios was less than the sum of individual effects of each scenario, indicating some redundancy between ART, VMMC, and increasing AFS in that age group.

Modeled mean AFS increased by 2 years in men and 3 years in women between 2000 and 2020 (Fig 6a), shifting the modeled age distribution of first sex to older ages (Fig 6b). Increases in AFS changed the modeled structure of the sexual network. Under the baseline scenario, where AFS was delayed, men of all ages formed fewer total sexual relationships due to fewer sexual partnerships available with younger women. However, in women, only younger women (<23 years of age) formed fewer relationships. Older women (23–31 years of age) formed *more* relationships, as men who were unable to form relationships with young women replaced those relationships with older women (Fig 6d).

## Discussion

Through retrospective analysis of cohort data and mathematical modeling, we found that increasing AFS (by 2–3 years between 2000 and 2019), ART scale-up, and VMMC scale-up had substantial impacts on reducing HIV incidence among AGYW. Increasing AFS was the largest contributor to incidence decline in women 15–19 years of age, whereas ART alone contributed the most to incidence decline in 20–24 years of age. The scale-up of VMMC impacted incidence over a longer time horizon, with a modest effect on historical incidence declines. These interventions and observed changes in sexual behavior, for the most part, acted additively, where their combined effects were equivalent to the sum of their independent effects. Our results suggest that sustained levels of ART, VMMC, and AFS will continue to reduce incidence in AGYW over the next 25–30 years.

Considerable investments have been made in behavioral and biomedical HIV prevention in AGYW [45,46]. The measured impact of these interventions on HIV risk in AGYW, however, has been mixed [47,48]. The effectiveness of PrEP for HIV prevention in women under age 25 has been suboptimal due to numerous barriers in product uptake and adherence [49–51]. The efficacy of oral PrEP measured in clinical trials, for example, underperformed expectations due to low adherence in AGYW [52,53]. Behavior change interventions have also demonstrated mixed effectiveness in promoting safer sexual behaviors and reducing HIV risk [54]. Sexual behavior in young adults is complex and often negotiated in the context of imbalanced social, economic, and gender-based power dynamics within partnerships, including the potential for coercion and gender-based violence, thus challenging interventions that target individual-level behaviors.

Despite the mixed effectiveness of behavioral interventions targeted at AGYW at high risk of HIV acquisition, long-term trends in SES and educational attainment may reduce social and behavioral vulnerability to HIV over their life course. Educational attainment is proximally associated with several behavioral risk factors including older age at sexual debut, older age at first marriage, fewer lifetime number of partners, lower probability of having an older male partner, lower frequency of sex with a partner, and less frequent unprotected sex [28,32,55–57]. Rising SES and lower prevalence of orphanhood, (resulting from an overall decline in HIV mortality), in the RCCS were associated with increases in school enrollment between 1994 and 2013, which in turn were associated with lower prevalence of HIV, lower adolescent pregnancy rates, higher use of modern contraception [58], and lower marriage rates [59], translating into increasing age at first marriage over time. The expansion of universal secondary education in Uganda in 2007 [60], which removed the barrier of school fees, may have also contributed to secular changes in sexual behavior, and, in turn, to declining HIV risk in our study [36]. Whether this trend is observed in other settings can add to an evidence base supporting expanded access to secondary education for young women as part of HIV prevention programs.

We found a disproportionately larger decline in incidence in women 15–24 years of age compared to older women, resulting in an age shift in incidence, a finding that has been confirmed from epidemiological [20] and phylogenetic data [21]. Reasons for age-heterogeneity in incidence decline are varied but include aging of HIV prevalence [20], targeted primary prevention to younger cohorts [61,62], and disproportionately larger reductions in sexual risk behaviors observed in younger cohorts [34]. Still, young adults in high-burden settings face challenges in achieving high coverage and effectiveness of treatment and prevention interventions that reduce risk in those populations. In our study, most men living with HIV under the age of 25 and nearly 40% of young women living with HIV (20–24 years of age) reported no ART use, even under universal test and treat, a pattern consistently observed across populations in sub-Saharan Africa [63]. Given the importance of ART as secondary prevention to reduce incidence in AGYW, targeted interventions towards increasing ART coverage in men, could contribute to additional reductions in HIV transmission [21].

There are several epidemiological and model-based assumptions that may limit the interpretation of our analysis. Self-reported sexual behavior is subject to social desirability bias, especially in the ‘Abstinence-First’ era in Uganda, which emerged in the early 2000’s as part of the abstinence, be faithful, use condoms approach, and may add some degree of error to our estimates of the proportion initiating sex in the population. Studies from East Africa have documented under-reporting of sexual activity in similar contexts [64]. Reporting bias alone, however, is unlikely to explain the large changes in AFS in our study. First, we observed a similar change in AFS in young men, who may experience less reporting pressures than their female peers. Second, abstinence-first programs have existed in Uganda prior to the start of the Rakai cohort, and thus any bias in reporting in the era following the introduction of abstinence-first would not be expected to change over time. Finally, national demographic indicators, such as rising age at marriage and first birth, corroborate real delays in sexual initiation [57]. We also rely on self-report of circumcision status and ART use to parameterize our model, both of which are subject to bias in level and trend over time, though previous analysis has also shown high specificity of self-reported ART when validated against clinically observed ART use [19].

In modeling delays in AFS over time, we assume that older male partners who would otherwise form relationships with younger women (who are delaying sex) are replacing those relationships with older women. This can, in turn, increase the number of older women forming relationships on the network, potentially offsetting some of the incidence reduction from young women delaying sex. Previous modeling has shown that the effects of delaying sexual debut are sensitive to whether partnership replacement is assumed [65]. It is plausible that the demand from men on the sexual network will draw more partners from similarly aged women as young women delay sex. In addition to commonly forming partnerships with younger women, a phenomenon observed consistently over time in the Rakai cohort [66] and elsewhere [27], older men also tend to partner with similarly aged women [67]. Further study is needed to understand how increasing AFS over time affects the structure of the sexual network, including any compensation for fewer young people available to form sexual relationships. These assumptions, however, likely result in a lower impact of delayed AFS in our model, and our findings should thus be interpreted as a conservative estimate of those effects.

Second, model-based incidence estimates for some years in our model tended to be slightly higher than cohort-based estimates, especially in the early years of the study period (pre-2010). The implications of this are that modeled changes in incidence over the study period may be overstated. Our model may not have fully captured the risk distribution of the study population earlier in the epidemic. One hypothesis is that higher-risk individuals have been systematically missing from the population from which incidence was estimated, especially in the earlier part of the study period. Incidence can only be directly observed in a resident population (who are present at multiple survey rounds). Mobile individuals, who tend to have higher HIV prevalence, would therefore be systematically excluded from the longitudinal cohort if they came into the cohort already positive. This would lead to a lower incidence in the population compared to our modeled estimates. Methods exist to correct for this bias by considering both left and right censoring using mechanistic modeling [68], though there are also limitations to this approach compared to directly observed methods. Furthermore, we projected estimated impacts of interventions/behavioral changes to 2050, and those estimates are subject to substantial uncertainty given future programmatic/funding shifts and other unforeseen dynamics.

Strengths of our analysis include the ability to disentangle the hypothesized drivers of HIV incidence decline in AGYW, providing evidence that existing interventions and behavioral changes act synergistically to impact both short and long-term incidence trajectories. We also dynamically model changes in sexual risk behavior over time to match observed longitudinal trends, something that previous models have only done theoretically [65]. Our model shows that both protective changes in risk behavior and scale-up of ART have contributed to historical declines in HIV incidence in AGYW (with VMMC acting over a longer time horizon), and if sustained, have the potential to continue to drive down infections in high-burden settings.

## Supporting information

S1 File **Table A. Incidence in the Rakai Community Cohort Study (RCCS) from 2000 to 2019.** Person-years (py), incident infections, and incidence (per 100 py) estimates and 95% confidence intervals (CI) using generalized linear models (GLMs) by age group, sex, and survey round (with corresponding year). **Table B. Model parameters used in calibration.** Median and interquartile range (IQR) from 100 best-fitting parameter sets. **Table C. Select static model parameters used to fit the EMOD HIV transmission model to Rakai survey data**. Models fit to population, HIV prevalence, and anti-retroviral therapy (ART) coverage. **Fig A. Modeled year-specific prevalence and 95% credible interval by sex for adults aged 15–49.** Modeled prevalence (red curve) fit to observed adult HIV prevalence in the Rakai cohort (black points with 95% confidence intervals). **Fig B. Modeled sex-, age-, and year-specific prevalence fit to observed prevalence in the Rakai cohort.** Point estimates and 95% credible intervals from model (lines with shaded ribbons) and observed prevalence (points and 95% confidence intervals). Note point estimates from Rakai cohort data are by round and thus are not available for some years.
(DOCX)

## Abbreviations

AGYW: adolescent girls and young women
ART: antiretroviral therapy
CIs: confidence intervals
GAMs: generalized additive models
GLMs: generalized linear models
PFA: pair formation algorithm
PrEP: pre-exposure prophylaxis
RCCS: Rakai Community Cohort Study
SES: socio-economic status
STIs: sexually transmitted infections
VMMC: voluntary medical male circumcision
