# Supplementary material for: Evaluating the biomedical and behavioral drivers of HIV incidence decline in adolescent girls and young women in Uganda: A mathematical modeling study
Source: PLoS Med. 2026 Mar 25;23(3):e1004993. doi: 10.1371/journal.pmed.1004993 (PMC13043050; doi:10.1371/journal.pmed.1004993)
Supplement: S1 File — Table A. Incidence in the Rakai Community Cohort Study (RCCS) from 2000 to 2019. Person-years (py), incident infections, and incidence (per 100 py) estimates and 95% confidence intervals (CI) using generalized linear models (GLMs) by age group, sex, and survey round (with corresponding year). Table B. Model parameters used in calibration. Median and interquartile range (IQR) from 100 best-fitting parameter sets. Table C. Select static model parameters used to fit the EMOD HIV transmission model to Rakai survey data. Models fit to population, HIV prevalence, and anti-retroviral therapy (ART) coverage. Fig A. Modeled year-specific prevalence and 95% credible interval by sex for adults aged 15–49. Modeled prevalence (red curve) fit to observed adult HIV prevalence in the Rakai cohort (black points with 95% confidence intervals). Fig B. Modeled sex-, age-, and year-specific prevalence fit to observed prevalence in the Rakai cohort. Point estimates and 95% credible intervals from model (lines with shaded ribbons) and observed prevalence (points and 95% confidence intervals). Note point estimates from Rakai cohort data are by round and thus are not available for some years. (DOCX) [file pmed.1004993.s001.docx]

Supplementary tables and figures:

Evaluating the biomedical and behavioural drivers of HIV incidence decline in adolescent girls and young women in Uganda: A mathematical modeling study

| Sex | Year | py | sero-conv | incidence (per 100py) | incidence (smoothed) | py | sero-conv | incidence (per 100py) | incidence (smoothed) | py | sero-conv | incidence (per 100py) | incidence (smoothed) |
| --- | --- | --- | --- | --- | --- | --- | --- | --- | --- | --- | --- | --- | --- |
| Women | 2000 | 1057 | 16 | 1.51 (0.93–2.47) | 1.57 (1.09–2.27) | 699 | 6 | 0.86 (0.39–1.91) | 1.35 (0.94–1.94) | 620 | 5 | 0.81 (0.34–1.94) | 0.81 (0.48–1.36) |
|  | 2001 | 1489 | 24 | 1.61 (1.08–2.40) | 1.60 (1.26–2.04) | 1177 | 19 | 1.61 (1.03–2.53) | 1.41 (1.08–1.84) | 915 | 7 | 0.76 (0.36–1.60) | 0.84 (0.57–1.24) |
|  | 2003 | 1570 | 25 | 1.59 (1.08–2.36) | 1.62 (1.31–2.00) | 1373 | 15 | 1.09 (0.66–1.81) | 1.46 (1.19–1.79) | 924 | 8 | 0.87 (0.43–1.73) | 0.87 (0.65–1.18) |
|  | 2004 | 1607 | 28 | 1.74 (1.20–2.52) | 1.60 (1.31–1.97) | 1589 | 30 | 1.89 (1.32–2.70) | 1.50 (1.26–1.78) | 1058 | 8 | 0.76 (0.38–1.51) | 0.91 (0.70–1.18) |
|  | 2005 | 1684 | 27 | 1.60 (1.10–2.34) | 1.55 (1.26–1.91) | 2107 | 33 | 1.57 (1.11–2.20) | 1.50 (1.28–1.75) | 1320 | 12 | 0.91 (0.52–1.60) | 0.93 (0.73–1.18) |
|  | 2007 | 1753 | 22 | 1.25 (0.83–1.91) | 1.49 (1.22–1.83) | 2681 | 35 | 1.31 (0.94–1.82) | 1.46 (1.25–1.70) | 1628 | 15 | 0.92 (0.56–1.53) | 0.93 (0.74–1.18) |
|  | 2009 | 1785 | 27 | 1.51 (1.04–2.21) | 1.44 (1.17–1.77) | 2896 | 44 | 1.52 (1.13–2.04) | 1.38 (1.19–1.61) | 1797 | 18 | 1.00 (0.63–1.59) | 0.91 (0.73–1.14) |
|  | 2010 | 1697 | 20 | 1.18 (0.76–1.83) | 1.39 (1.13–1.71) | 2669 | 36 | 1.35 (0.97–1.87) | 1.28 (1.10–1.49) | 1804 | 23 | 1.27 (0.85–1.92) | 0.85 (0.68–1.06) |
|  | 2012 | 1848 | 26 | 1.41 (0.96–2.07) | 1.30 (1.06–1.61) | 2761 | 31 | 1.12 (0.79–1.60) | 1.15 (0.99–1.34) | 2001 | 14 | 0.70 (0.41–1.18) | 0.76 (0.61–0.95) |
|  | 2014 | 2201 | 29 | 1.32 (0.92–1.90) | 1.14 (0.91–1.41) | 3367 | 27 | 0.80 (0.55–1.17) | 1.02 (0.87–1.20) | 2778 | 15 | 0.54 (0.33–0.90) | 0.66 (0.54–0.82) |
|  | 2016 | 2072 | 23 | 1.11 (0.74–1.67) | 0.89 (0.70–1.13) | 2773 | 34 | 1.23 (0.88–1.72) | 0.89 (0.74–1.07) | 2687 | 12 | 0.45 (0.25–0.79) | 0.58 (0.46–0.73) |
|  | 2017 | 2065 | 7 | 0.34 (0.16–0.71) | 0.64 (0.47–0.87) | 2633 | 20 | 0.76 (0.49–1.18) | 0.76 (0.61–0.96) | 2831 | 16 | 0.57 (0.35–0.92) | 0.51 (0.39–0.67) |
|  | 2019 | 1999 | 9 | 0.45 (0.23–0.87) | 0.45 (0.28–0.72) | 2690 | 13 | 0.48 (0.28–0.83) | 0.65 (0.47–0.88) | 3405 | 16 | 0.47 (0.29–0.77) | 0.45 (0.32–0.65) |
|  | Total | 22827 | 283 | 1.24 (1.10–1.39) |  | 29415 | 343 | 1.17 (1.05–1.30) | | 23768 | 169 | 0.71 (0.61–0.83) |  |
|  |  |  |  |  |  |  |  |  |  |  |  |  |  |
| Men | 2000 | 776 | 9 | 1.16 (0.60–2.23) | 1.27 (0.90–1.79) | 684 | 11 | 1.61 (0.89–2.90) | 1.46 (1.00–2.14) | 380 | 1 | 0.26 (0.04–1.87) | 0.96 (0.53–1.74) |
|  | 2001 | 1193 | 10 | 0.84 (0.45–1.56) | 1.13 (0.85–1.50) | 1077 | 11 | 1.02 (0.57–1.84) | 1.53 (1.17–2.01) | 617 | 10 | 1.62 (0.87–3.01) | 0.98 (0.63–1.52) |
|  | 2003 | 1267 | 16 | 1.26 (0.77–2.06) | 1.00 (0.79–1.27) | 1229 | 19 | 1.55 (0.99–2.42) | 1.61 (1.31–1.99) | 719 | 5 | 0.70 (0.29–1.67) | 0.99 (0.71–1.40) |
|  | 2004 | 1307 | 12 | 0.92 (0.52–1.62) | 0.89 (0.72–1.10) | 1364 | 27 | 1.98 (1.36–2.89) | 1.67 (1.39–2.01) | 812 | 8 | 0.99 (0.49–1.97) | 0.99 (0.74–1.33) |
|  | 2005 | 1366 | 9 | 0.66 (0.34–1.27) | 0.78 (0.64–0.96) | 1716 | 31 | 1.81 (1.27–2.57) | 1.67 (1.41–1.99) | 1090 | 10 | 0.92 (0.49–1.71) | 0.98 (0.75–1.28) |
|  | 2007 | 1547 | 11 | 0.71 (0.39–1.28) | 0.69 (0.56–0.84) | 2071 | 27 | 1.30 (0.89–1.90) | 1.62 (1.37–1.91) | 1401 | 11 | 0.79 (0.43–1.42) | 0.94 (0.73–1.21) |
|  | 2009 | 1776 | 12 | 0.68 (0.38–1.19) | 0.60 (0.49–0.74) | 2159 | 43 | 1.99 (1.48–2.69) | 1.50 (1.27–1.77) | 1633 | 16 | 0.98 (0.60–1.60) | 0.87 (0.68–1.12) |
|  | 2010 | 1728 | 16 | 0.93 (0.57–1.51) | 0.52 (0.42–0.65) | 1966 | 21 | 1.07 (0.70–1.64) | 1.33 (1.11–1.58) | 1680 | 19 | 1.13 (0.72–1.77) | 0.76 (0.59–0.98) |
|  | 2012 | 1873 | 8 | 0.43 (0.21–0.85) | 0.45 (0.36–0.57) | 2046 | 24 | 1.17 (0.79–1.75) | 1.13 (0.94–1.35) | 1839 | 11 | 0.60 (0.33–1.08) | 0.62 (0.48–0.81) |
|  | 2014 | 2373 | 5 | 0.21 (0.09–0.51) | 0.39 (0.30–0.50) | 2552 | 27 | 1.06 (0.73–1.54) | 0.93 (0.76–1.13) | 2608 | 9 | 0.35 (0.18–0.66) | 0.49 (0.38–0.64) |
|  | 2016 | 2265 | 5 | 0.22 (0.09–0.53) | 0.33 (0.25–0.45) | 2125 | 12 | 0.56 (0.32–0.99) | 0.74 (0.59–0.93) | 2388 | 10 | 0.42 (0.23–0.78) | 0.38 (0.28–0.51) |
|  | 2017 | 2329 | 6 | 0.26 (0.12–0.57) | 0.29 (0.20–0.41) | 2054 | 13 | 0.63 (0.37–1.09) | 0.59 (0.44–0.78) | 2453 | 7 | 0.29 (0.14–0.60) | 0.29 (0.20–0.43) |
|  | 2019 | 2262 | 7 | 0.31 (0.15–0.65) | 0.25 (0.16–0.37) | 2121 | 8 | 0.38 (0.19–0.75) | 0.46 (0.31–0.69) | 2625 | 5 | 0.19 (0.08–0.46) | 0.22 (0.13–0.38) |
|  | Total | 22062 | 126 | 0.57 (0.48–0.68) |  | 23164 | 274 | 1.18 (1.05–1.33) | | 20245 | 122 | 0.60 (0.50–0.72) |  |

**Table A.**  **Incidence in the Rakai Community Cohort Study (RCCS) from 2000 – 2019**

Person-years (py), incident infections, and incidence (per 100 py) estimates and 95% confidence intervals (CI) using generalized linear models (GLMs) by age group, sex, and survey round (with corresponding year).

|  | Parameter value quantile | | |
| --- | --- | --- | --- |
|  | 25% | 50% | 75% |
| Acute duration (months) | 3.99 | 4.24 | 4.70 |
| Acute stage multiplier on base infectivity | 10 | 10 | 18 |
| Base infectivity | 0.001142 | 0.001253 | 0.00141 |
| Circumcision efficacy | 0.62 | 0.66 | 0.69 |
| Initial proportion of low-risk individuals | 0.550 | 0.622 | 0.636 |
| Initial seeding proportion of infections into high-risk group (proportion) | 0.09 | 0.10 | 0.10 |
| Male to female infectivity multiplier for women 25 years and older | 1.62 | 1.81 | 2.21 |
| Male to female infectivity multiplier for women < 25 years | 3.58 | 4.59 | 6.36 |
| Mean drop out after ART initiation (days) | 1293 | 1324 | 1661 |
| Year HIV infections are seeded into high-risk group | 1980 | 1981 | 1983 |
| **Table B**. **Model parameters used in calibration.**  Median and interquartile range (IQR) from 100 best-fitting parameter sets values |  |  |  |
|  |  |  |  |
|  |  |  |  |
|  |  |  |  |
|  |  |  |  |
|  |  |  |  |
|  |  |  |  |
|  |  |  |  |

| Parameter | Description | Value / Median (IQR) |
| --- | --- | --- |
| AIDS_Duration_In_Months | The length of time, in months, prior to an AIDS-related death over which the AIDS_Stage_Infectivity_Multiplier is applied | 9 |
| AIDS_Stage_Infectivity_Multiplier | Multiplier acting on Base_Infectivity to determine the per-act transmission probability of an individual during AIDS stage | 4.5 |
| ART_CD4_at_Initiation_Saturating_Reduction_in_Mortality | The duration from ART enrollment to on-ART HIV-caused death increases with CD4 at ART initiation up to a threshold determined by this parameter value. | 350 |
| ART_Viral_Suppression_Multiplier | Multiplier acting on Base_Infectivity to determine the per-act transmission probability of a virally suppressed HIV+ individual. | 0.08 |
| CD4_At_Death_LogLogistic_Heterogeneity | The inverse shape parameter of a Weibull distribution that represents the at-death CD4 cell count. | 0.7 |
| CD4_At_Death_LogLogistic_Scale | The scale parameter of a Weibull distributionthat represents the at-death CD4 cell count. | 2.96 |
| CD4_Post_Infection_Weibull_Heterogeneity | The inverse shape parameter of a Weibull distributionthat represents the post-acute-infection CD4 cell count. | 0.2756 |
| CD4_Post_Infection_Weibull_Scale | The scale parameter of a Weibull distributionthat represents the post-acute-infection CD4 cell count. | 560.43 |
| Coital_Act_Rate | Number of coital acts per day for all relatinoships except commercial ones | 0.33 |
| Coital_Act_Rate_Commercial | Number of coital acts per day for commercial relationships | 0.002739726 |
| Coital_Dilution_Factor_2_Partners | The multiplicative reduction in the coital act rate for all relationship types when an individual has exactly two current partners. Represents coital dilution. | 0.75 |
| Coital_Dilution_Factor_3_Partners | The multiplicative reduction in the coital act rate for all relationship types when an individual has exactly three current partners. Represents coital dilution. | 0.6 |
| Coital_Dilution_Factor_4_Plus_Partners | The multiplicative reduction in the coital act rate for all relationship types when an individual has exactly three current partners. Represents coital dilution. | 0.45 |
| Commercial_Condom_Max | The maximum asymptote for commercial relationships | 0.85 |
| Commercial_Condom_Mid | The year of the inflection point for commercial relationships | 1999.5 |
| Commercial_Condom_Min | The minimum asymptote of the probability of condom use per coital act for informal relationships for commercial relationships | 0.5 |
| Commercial_Condom_Rate | The rate proportional to the slope at the inflection point for commercial relationships | 1 |
| Commercial_Form_Rate | Exponentially distributed mean number new relationships formed per day for commercial relationships | 0.15 |
| Condom_Transmission_Blocking_Probability | The per-act multiplier of the transmission probability when a condom is used | 0.8 |
| Days_Between_Symptomatic_And_Death_Weibull_Heterogeneity | The time between the onset of AIDS symptoms and death is sampled from a Weibull distribution; this parameter governs the heterogeneity (inverse shape) of the Weibull. | 0.5 |
| Days_Between_Symptomatic_And_Death_Weibull_Scale | The time between the onset of AIDS symptoms and death is sampled from a Weibull distribution; this parameter governs the scale of the Weibull. | 618.341625 |
| HIV_Adult_Survival_Scale_Parameter_Intercept | Determines the intercept of the scale parameter for the Weibull distribution used to determine HIV survival time. Survival time with untreated HIV infection depends on the age of the individual at the time of infection, and is drawn from a Weibull distribution with shape parameter (see HIV_Adult_Survival_Shape_Parameter) and scale parameter. The scale parameter is allowed to vary linearly with age as follows λ = HIV_Adult_Survival_Scale_Parameter_Intercept + HIV_Adult_Survival_Scale_ Parameter_Slope * Age (in years). | 21.182 |
| HIV_Adult_Survival_Scale_Parameter_Slope | This parameter determines the slope of the scale parameter for the Weibull distribution used to determine HIV survival time. | -0.2717 |
| HIV_Adult_Survival_Shape_Parameter | This parameter determines the shape of the Weibull distribution used to determine age-dependent survival time for individuals infected with HIV. | 2 |
| HIV_Age_Max_for_Adult_Age_Dependent_Survival | Survival time with untreated HIV infection depends on the age of the individual at the time of infection, and is drawn from a Weibull distribution with shape parameter and scale parameters (See HIV_Adult_Survival_Scale_Parameter_Intercept, HIV_Adult_Survival_Scale_ Parameter_Slope, and HIV_Adult_Survival_Shape_Parameter). Although the scale parameter for survival time declines with age, it cannot become negative. To avoid negative survival times at older ages, this parameter, HIV_Age_Max_for_Adult_Age_Dependent_Survival, determines the age beyond which HIV survival is no longer affected by further aging. | 50 |
| HIV_Age_Max_for_Child_Survival_Function | The maximum age at which an individual’s survival will be fit to the child survival function. If the value of this parameter falls between zero and the age of sexual debut, model results are not sensitive to this parameter as there is no mechanism for children to become infected between infancy and sexual debut. | 15 |
| HIV_Child_Survival_Rapid_Progressor_Fraction | The proportion of HIV-infected children who are rapid HIV progressors. | 0.57 |
| HIV_Child_Survival_Rapid_Progressor_Rate | The exponential decay rate, in years, describing the distribution of HIV survival for children who are rapid progressors. | 1.52 |
| HIV_Child_Survival_Slow_Progressor_Scale | The Weibull scale parameter describing the distribution of HIV survival for children who are slower progressors. | 16 |
| HIV_Child_Survival_Slow_Progressor_Shape | The Weibull shape parameter describing the distribution of HIV survival for children who are slower progressors. | 2.7 |
| Maternal_Infection_Transmission_Probability | The probability of transmission of infection from mother to infant at birth. | 0.3 |
| Maternal_Transmission_ART_Multiplier | The maternal transmission multiplier for on-ART mothers. | 0.03334 |
| Sexual_Debut_Age_Min | The minimum age at which individuals become eligible to form sexual relationships. | 13 |

**Table C. Select static model parameters used to fit the EMOD HIV transmission model to Rakai survey data**,

Models fit to population, HIV prevalence, and anti-retroviral therapy (ART) coverage.


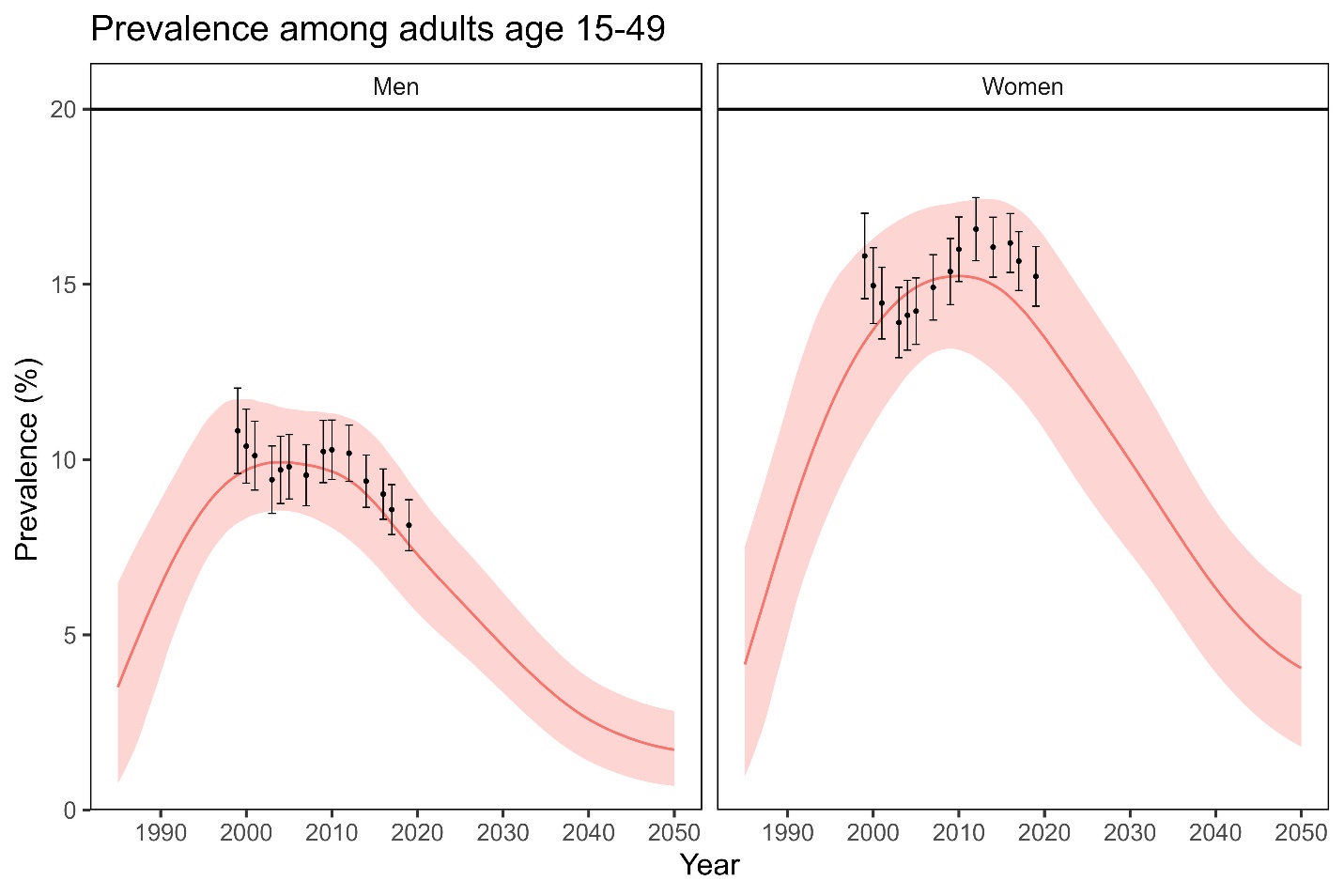


**Fig A.** **Modeled year-specific prevalence and 95% credible interval by sex for adults aged 15–49**

Modeled prevalence (red curve) fit to observed adult HIV prevalence in the Rakai cohort (black points with 95% confidence intervals).


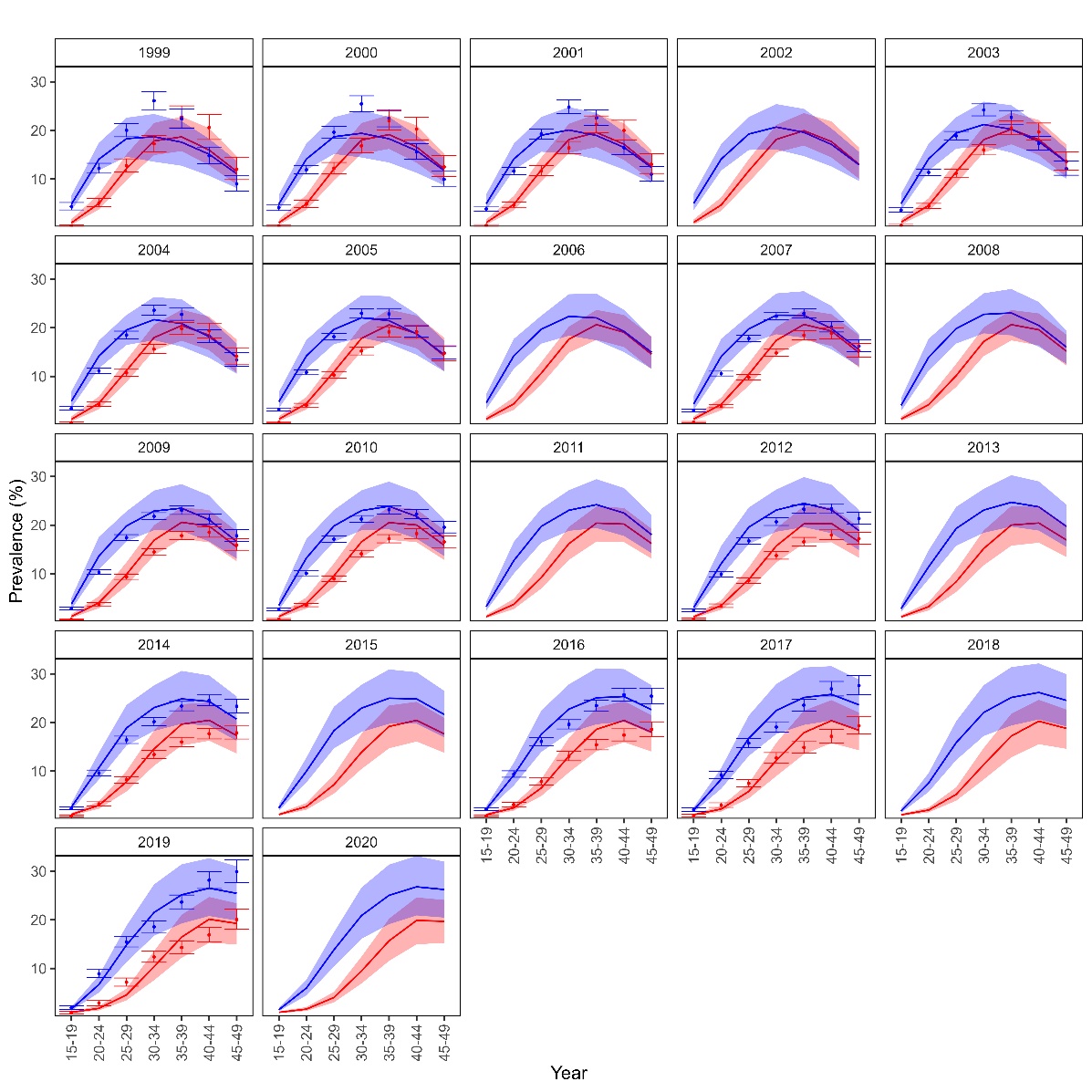


**Fig B. Modeled sex-, age-, and year-specific prevalence fit to observed prevalence in the Rakai cohort**

Point estimates and 95% credible intervals from model (lines with shaded ribbons) and observed prevalence (points and 95% confidence intervals). Note point estimates from Rakai cohort data are by round and thus are not available for some years.
